# Vaccination Coverage by Age 24 Months Among Children Born During 2017–2021 — U.S.-Affiliated Pacific Islands

**DOI:** 10.15585/mmwr.mm7338a4

**Published:** 2024-09-26

**Authors:** Ashley Tippins, E.M. Boyd, Kelsey C. Coy, Glodi Mutamba, Jennifer L. Kriss

**Affiliations:** ^1^Immunization Services Division, National Center for Immunization and Respiratory Diseases, CDC; ^2^Eagle Health Analytics, San Antonio, Texas.

**Summary**

**What is already known about this topic?**

Childhood vaccination is one of the most successful public health interventions. The U.S.-affiliated Pacific Islands (USAPI) immunization programs and CDC collaborate to monitor vaccination coverage in their jurisdictions.

**What is added by this report?**

This report is the first comprehensive analysis of trends in childhood vaccination coverage in USAPI. Coverage of >90% by age 24 months with recommended vaccines was inconsistently met across jurisdictions.

**What are the implications for public health practice?**

Gaps in coverage identified in this report can be used to identify where evaluation of jurisdiction-specific reasons for lagging on-time vaccination is needed. Monitoring of vaccination coverage can be used to guide intervention implementation and evaluate effectiveness of interventions.

## Abstract

Childhood vaccination is one of the most successful public health interventions to improve life expectancy, decrease health care costs, and reduce the spread of preventable diseases. Using data from jurisdictional immunization information systems, vaccination coverage by age 24 months among children born during 2017–2021 in the U.S.-affiliated Pacific Islands was estimated for all vaccines included in jurisdictional programs. Progress toward the U.S. Healthy People 2030 and World Health Organization Immunization Agenda 2030 vaccination goals of ≥90% coverage by age 24 months with recommended vaccines was inconsistently met across jurisdictions. For example, coverage by age 24 months with ≥1 dose of measles, mumps, and rubella vaccine ranged from 68.2% to 91.6% by birth cohort in Federated States of Micronesia and from 87.4% to 96.6% in Palau; coverage with ≥4 doses of diphtheria and tetanus toxoids and acellular pertussis vaccine (DTaP) ranged from 39.6% to 60.6% in Federated States of Micronesia and from 73.4% to 85.4% in Palau. Coverage as of June 1, 2024, increased for all vaccines across all jurisdictions and birth cohorts, indicating catch-up vaccination after age 24 months. For example, coverage with ≥4 doses of DTaP by June 1, 2024, ranged from 74.0% to 84.4% in American Samoa by birth cohort and from 91.6% to 94.8% in Palau. This report is the first comprehensive analysis of trends in childhood vaccination coverage in the U.S.-affiliated Pacific Islands; data in this report can be used to determine where additional efforts are needed to assess reasons for delayed vaccination of children and strategies to mitigate vaccination delays, specific to each jurisdiction.

## Introduction

Childhood vaccination is one of the most successful public health interventions to improve life expectancy, decrease health care costs, and reduce the spread of preventable diseases (*1*). The U.S Healthy People 2030 and the World Health Organization (WHO) Immunization Agenda 2030 have established 90% vaccination targets* for the following childhood vaccines: diphtheria and tetanus toxoids and acellular pertussis vaccine (DTaP); measles, mumps, and rubella vaccine (MMR); and pneumococcal conjugate vaccine (PCV) (*2*,*3*).

All six of the U.S.-affiliated Pacific Islands^†^ (USAPI) participate in the U.S. domestic immunization program. CDC collaborates with USAPI immunization programs to monitor vaccination coverage with all vaccines included in jurisdictional programs. This report describes vaccination coverage by age 24 months in five of the six USAPI jurisdictions^§^ among children born during 2017–2021. 

## Methods

### Data Sources and Inclusion and Exclusion Criteria

Patient-level data from jurisdictional immunization information systems (IISs) were aggregated at the jurisdiction level. Patients were grouped by calendar year of birth (i.e., birth cohort) and were included in the denominator if they had an active patient status^¶^ in IIS. Patients with an inactive or deceased status were excluded from all analyses. To mitigate potential denominator inflation, patients with no vaccine doses recorded in IIS were also excluded, consistent with the Modeling of Immunization Registry Operations Workgroup’s guidance for assessment at the jurisdiction level (*4*).

### Estimation of Vaccination Coverage

Coverage estimates included all vaccine doses received as of the day before each child reached age 24 months (i.e., on-time vaccination) so that coverage by age 24 months could be assessed, with the exception of rotavirus vaccine, which is assessed by age 8 months because of the upper age limit for receipt of this vaccine. Coverage estimates included the following vaccines: DTaP, poliovirus, MMR, *Haemophilus influenzae* type b (Hib), hepatitis B (HepB), PCV, rotavirus, and the combined six-vaccine series (4:3:1:3*:3:4).** Hepatitis A vaccine (HepA) and varicella vaccine were included for jurisdictions where these vaccines have been introduced into the vaccination program, and for 2 doses of MMR in jurisdictions where 2 doses are recommended by age 24 months.^††^ To assess catch-up vaccination after age 24 months, all doses received as of June 1, 2024, were included in coverage estimates. SAS software (version 9.4; SAS Institute) was used to conduct all analyses. This activity was reviewed by CDC, deemed not research, and was conducted consistent with applicable federal law and CDC policy.^§§^

## Results

### Jurisdictional Vaccination Coverage by Age 24 Months

Coverage by age 24 months was ≥90% among one or more birth cohorts with each of the following vaccines: ≥3 doses of DTaP, ≥3 doses of poliovirus vaccine, and ≥3 doses of HepB (Northern Mariana Islands and Palau), ≥1 dose MMR and Hib primary series^¶¶^ (Northern Mariana Islands, Federated States of Micronesia, and Palau); HepB birth dose*** (American Samoa, Northern Mariana Islands, Marshall Islands, and Palau); and ≥3 doses of PCV (Palau) (Table 1). Coverage with ≥4 doses of DTaP, the Hib full series, ≥4 PCV doses, and rotavirus (by age 8 months) was <90% in all jurisdictions.^†††^ Coverage with the 4:3:1:3*:3:4 series was <75% across all jurisdictions. Coverage with HepA and varicella vaccine among the U.S. territories (American Samoa and Northern Mariana Islands), and with ≥2 doses of MMR among the freely associated states (Federated States of Micronesia, Marshall Islands, and Palau) was <90% (Supplementary Table, https://stacks.cdc.gov/view/cdc/162211).

**TABLE 1:** Estimated vaccination coverage with selected vaccines and vaccine series by age 24 months among children born during 2017–2021,* by jurisdiction and year of birth — U.S.-affiliated Pacific Islands

Jurisdiction/Birth year (n)	%											
	DTaP		Poliovirus ≥3 doses	MMR ≥1 dose	Hib^†^		HepB		PCV		Rotavirus^¶^	Combined six-vaccine series (4:3:1:3*:3:4)**
	≥3 doses	≥4 doses			Primary series	Full series	Birth dose^§^	≥3 doses	≥3 doses	≥4 doses		
**American Samoa**												
2017 (1,254)	79.2	52.0	77.4	70.4	70.3	25.0	94.6	76.6	71.1	49.5	5.2	18.3
2018 (1,162)	80.1	53.8	78.5	85.1	73.8	34.9	93.3	70.6	72.6	52.5	3.7	26.0
2019 (1,028)	81.4	53.5	80.0	81.5	72.9	29.0	87.7	78.8	73.7	54.6	3.7	21.7
2020 (860)	84.9	51.9	84.1	77.2	78.4	33.4	96.4	86.6	74.3	54.8	4.5	26.3
2021 (832)	81.5	53.4	80.4	82.7	72.7	31.4	96.9	84.3	70.1	47.7	4.9	23.8
**Northern Mariana Islands**												
2017 (715)	87.8	67.3	87.3	88.7	84.1	51.9	89.7	91.0	84.8	62.9	53.7	46.3
2018 (723)	92.1	74.0	91.4	90.5	90.5	65.7	94.1	92.9	89.6	68.7	60.4	60.0
2019 (746)	92.0	71.3	91.6	87.0	92.4	71.0	95.0	89.5	89.5	67.2	68.0	64.6
2020 (651)	92.0	68.0	91.4	86.5	92.2	68.7	94.3	92.8	89.2	66.2	72.0	61.8
2021 (603)	86.1	66.8	85.4	83.1	86.4	65.0	95.9	88.9	83.6	62.9	60.9	60.0
**Federated States of Micronesia**												
2017 (2,139)	77.5	50.5	77.0	83.2	87.6	66.8	71.9	83.9	70.4	42.9	31.8	37.4
2018 (2,097)	85.6	60.6	85.1	91.6	91.3	77.2	73.2	89.1	81.7	54.6	37.1	49.3
2019 (1,920)	79.0	45.6	79.0	76.0	89.2	67.3	72.8	84.9	77.4	48.1	40.9	37.7
2020 (1,906)	69.5	39.6	69.2	68.2	81.0	56.0	66.3	74.9	66.7	40.5	42.9	31.5
2021 (1,855)	75.7	45.7	75.3	84.7	85.1	65.2	76.3	80.7	71.4	46.1	38.7	37.3
**Marshall Islands**												
2017 (1,074)	74.9	55.9	74.5	78.3	68.3	4.6	82.1	79.7	60.1	32.7	41.9	2.2
2018 (1,142)	79.6	63.9	79.3	88.7	75.4	6.6	69.9	84.1	61.9	35.6	45.7	3.4
2019 (1,072)	82.2	62.4	81.6	84.4	74.9	3.5	87.8	87.1	63.2	33.3	49.9	1.8
2020 (1,004)	86.1	61.9	85.7	82.7	72.4	3.8	87.0	89.0	60.9	31.5	54.5	1.1
2021 (994)	84.5	58.4	84.2	84.9	69.2	4.2	90.0	87.9	60.6	26.0	45.1	1.6
**Palau**												
2017 (232)	92.7	76.7	93.5	89.2	91.8	81.5	94.0	94.0	83.6	67.7	72.4	61.6
2018 (268)	95.9	85.4	95.1	95.1	96.6	89.9	96.6	96.3	90.3	76.9	82.1	72.4
2019 (234)	94.0	78.6	94.0	89.3	96.2	88.5	94.0	95.3	91.9	75.6	80.3	69.2
2020 (214)	92.5	73.4	92.5	87.4	95.3	85.5	95.8	93.0	89.3	79.9	82.7	69.6
2021 (206)	97.1	84.5	97.1	96.6	98.5	89.3	98.1	98.1	93.7	67.0	55.3	61.7

**Abbreviations:** DTaP = diphtheria, tetanus toxoids, and acellular pertussis vaccine; HepB = hepatitis B vaccine; Hib = *Haemophilus influenzae *type b conjugate vaccine; MMR = measles, mumps, and rubella vaccine; PCV = pneumococcal conjugate vaccine.

* Vaccination status includes vaccine doses received by age 24 months (i.e., before the day the child turns age 24 months), except for the HepB birth dose and rotavirus vaccination by age 8 months. The denominator includes patients with an active patient status in an immunization information system as of June 1, 2024. Patient active status in an immunization information system establishes a classification of individual patients within a health care organization. Health care providers are responsible for vaccinating patients with an active status within their clinic population or geographic catchment area. Patient status is changed to inactive when the patient changes providers, moves, or is lost to follow-up, or deceased if patient death is confirmed through manual review or system linkage with vital statistics or other health records. https://repository.immregistries.org/files/resources/5835adc2dad8d/mirow_pais_mini-guide.pdf

^†^ Hib primary series = receipt of ≥2 or ≥3 doses, depending on product type received; full series = primary series and booster dose, which includes receipt of ≥3 or ≥4 doses, depending on product type received.

^§^ One dose of HepB administered from birth through age 3 days.

^¶^ Includes ≥2 doses of Rotarix monovalent rotavirus vaccine (GSK) or ≥3 doses of RotaTeq pentavalent rotavirus vaccine (Merck and Co., Inc.); if any dose in the series is either RotaTeq or unknown, the default is to a 3-dose series. The maximum age for the final rotavirus dose is 8 months, 0 days.

** The combined six-vaccine series (4:3:1:3*:3:4) includes ≥4 doses of DTaP, ≥3 doses of poliovirus vaccine, ≥1 dose of measles-containing vaccine, the full Hib series (≥3 or ≥4 doses, depending on product type), ≥3 doses of HepB, and ≥4 doses of PCV.

### Trends in Vaccination Coverage by Birth Cohort

Vaccination coverage by age 24 months fluctuated by birth cohort across jurisdictions; for example, coverage by age 24 months with ≥1 dose of MMR ranged from 68.2% to 91.6% in Federated States of Micronesia by birth cohort and from 87.4% to 96.6% in Palau; coverage with ≥4 doses of DTaP ranged from 39.6% to 60.6% in Federated States of Micronesia and from 73.4% to 85.4% in Palau (Table 1). Across all jurisdictions and for most vaccines, coverage was higher among children born in 2018 than among those born in 2017. Coverage with most vaccines was lower among children born in 2019 and 2020 than among those born in 2018. Compared with children born in 2020, among those born in 2021, coverage with some vaccines began to increase. For example, coverage with MMR was 5.5 percentage points higher in American Samoa, 16.5 percentage points higher in Federated States of Micronesia, and 9.2 percentage points higher in Palau; however, the magnitude and direction of coverage varied across vaccines and jurisdictions.

Coverage with all vaccines increased across all jurisdictions and birth cohorts when vaccination status was assessed as of June 1, 2024, indicating catch-up vaccination after age 24 months. For example, among the 2021 birth cohort, coverage with ≥4 doses of DTaP (Palau), ≥3 doses of poliovirus vaccine (Marshall Islands), ≥1 dose of MMR (American Samoa, Federated States of Micronesia, and Marshall Islands), ≥3 doses of HepB (Northern Mariana Islands, Federated States of Micronesia, and Marshall Islands), and the full Hib series (Palau) increased from <90% by age 24 months to ≥90% as of June 1, 2024 (Figure). Coverage increases by June 1, 2024, were highest for ≥4 doses of DTaP (range = 9.2–20.7 percentage points); coverage with ≥4 doses of DTaP by June 1, 2024, ranged from 74.0% to 84.4% by birth cohort in American Samoa and from 91.6% to 94.8% in Palau (Table 2).

**FIGURE:**
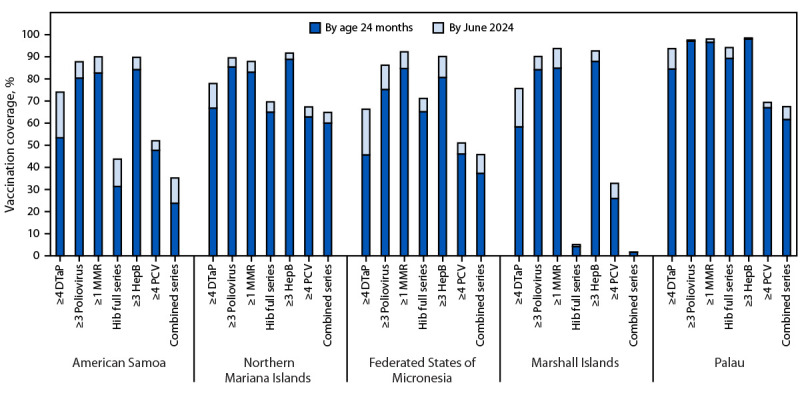
Estimated vaccination coverage with selected individual vaccines* and a combined vaccine series^†^ by age 24 months^§^ and catch-up coverage among children born in 2021, by jurisdiction — U.S.-affiliated Pacific Islands, June 1, 2024^¶^ **Abbreviations:** DTaP = diphtheria, tetanus toxoids, and acellular pertussis vaccine; HepB = hepatitis B vaccine; Hib = *Haemophilus influenzae* type b conjugate vaccine; MMR = measles, mumps, and rubella vaccine; PCV = pneumococcal conjugate vaccine. * Hib primary series = receipt of ≥2 or ≥3 doses, depending on product type received; full series = primary series and booster dose, which includes receipt of ≥3 or ≥4 doses, depending on product type received. ^†^ The combined six-vaccine series (4:3:1:3*:3:4) includes ≥4 doses of DTaP, ≥3 doses of poliovirus vaccine, ≥1 dose of measles-containing vaccine, the full series of Hib (≥3 or ≥4 doses, depending on product type), ≥3 doses of HepB, and ≥4 doses of PCV. ^§^ Vaccination status includes vaccine doses received by age 24 months (i.e., before the day the child turns age 24 months). The denominator includes patients with an active patient status in an immunization information system as of June 1, 2024. Patient active status in the immunization information system establishes a classification of individual patients within a health care organization. Health care providers are responsible for vaccinating patients with an active status within their clinic population or geographic catchment area. Patient status is changed to inactive when the patient changes providers, moves, or is lost to follow-up, or deceased if patient death is confirmed through manual review or system linkage with vital statistics or other health records. https://repository.immregistries.org/files/resources/5835adc2dad8d/mirow_pais_mini-guide.pdf ^¶^ Vaccination status includes all vaccine doses received by June 1, 2024. The denominator includes patients with an active patient status in an immunization information system as of June 1, 2024. Patient active status in an immunization information system establishes a classification of individual patients within a health care organization. Health care providers are responsible for vaccinating patients with an active status within their clinic population or geographic catchment area. Patient status is changed to inactive when the patient changes providers, moves, or is lost to follow-up, or deceased if patient death is confirmed through manual review or system linkage with vital statistics or other health records. https://repository.immregistries.org/files/resources/5835adc2dad8d/mirow_pais_mini-guide.pdf

**TABLE 2:** Estimated vaccination coverage with selected vaccines and vaccine series among children born during 2017–2021,* by jurisdiction and year of birth — U.S.-affiliated Pacific Islands, June 2024

Jurisdiction/Birth year (n)	%									
	DTaP		Poliovirus ≥3 doses	MMR ≥1 dose	Hib^†^		HepB (≥3 doses)	PCV		Combined six-vaccine series (4:3:1:3*:3:4)**
	≥3 doses	≥4 doses			Primary series	Full series		≥3 doses	≥4 doses	
**American Samoa**										
2017 (1,254)	88.4	82.0	88.0	86.4	78.9	41.1	87.4	76.6	57.7	33.8
2018 (1,162)	89.8	82.4	88.7	89.4	80.6	50.7	88.4	78.3	59.3	43.9
2019 (1,028)	87.7	82.1	87.0	90.5	83.2	46.7	87.8	79.3	62.2	39.0
2020 (860)	93.5	84.4	93.4	92.0	88.5	55.5	93.7	81.4	64.5	45.8
2021 (832)	88.2	74.0	87.7	90.0	78.6	43.8	89.8	75.4	52.0	35.2
**Northern Mariana Islands**										
2017 (715)	97.2	95.5	96.9	98.2	89.7	59.2	97.6	90.2	70.6	53.4
2018 (723)	96.8	93.4	96.8	96.0	92.3	71.5	97.1	92.9	73.7	65.8
2019 (746)	95.6	89.5	94.9	94.2	94.5	82.7	96.5	92.6	78.4	77.1
2020 (651)	95.5	85.7	95.2	92.6	94.2	78.3	95.9	92.6	76.3	73.3
2021 (603)	89.6	77.9	89.6	87.9	89.4	69.7	91.7	87.4	67.3	64.8
**Federated States of Micronesia**										
2017 (2,139)	94.6	91.1	94.3	96.1	91.7	79.1	95.0	82.8	55.6	54.7
2018 (2,097)	95.4	91.1	95.3	97.9	93.3	82.1	96.7	87.2	60.2	59.3
2019 (1,920)	93.2	82.1	92.7	94.9	93.9	82.8	94.9	87.3	59.2	56.5
2020 (1,906)	88.9	74.8	88.6	93.6	88.7	72.6	91.1	78.9	54.2	50.5
2021 (1,855)	86.5	66.3	86.2	92.2	89.1	71.2	90.1	77.6	51.1	45.8
**Marshall Islands**										
2017 (1,074)	93.0	89.7	92.7	93.7	85.4	6.9	92.3	69.0	41.7	3.2
2018 (1,142)	95.3	90.7	94.9	95.3	87.4	8.1	94.7	68.6	40.6	4.2
2019 (1,072)	94.7	89.7	94.3	95.2	87.2	5.5	94.9	71.5	39.2	2.4
2020 (1,004)	94.1	87.3	93.9	96.2	85.9	5.5	94.5	70.6	37.7	2.0
2021 (994)	90.9	75.7	90.1	93.8	81.7	5.1	92.7	65.2	32.8	1.7
**Palau**										
2017 (232)	96.6	94.8	97.0	97.0	94.0	83.2	97.0	88.8	71.6	66.8
2018 (268)	98.5	93.7	98.5	97.0	97.0	90.7	98.5	90.7	78.0	76.1
2019 (234)	97.0	94.0	97.0	96.6	97.9	91.9	97.9	94.9	81.2	79.1
2020 (214)	95.8	91.6	96.3	96.3	97.7	91.6	95.8	92.5	85.5	80.8
2021 (206)	97.6	93.7	97.6	98.1	98.5	94.2	98.5	95.6	69.4	67.5

**Abbreviations:** DTaP = diphtheria, tetanus toxoids, and acellular pertussis vaccine; HepB = hepatitis B vaccine; Hib = *Haemophilus influenzae* type b conjugate vaccine; MMR = measles, mumps, and rubella vaccine; PCV = pneumococcal conjugate vaccine.

* Vaccination status includes all vaccine doses received by June 1, 2024. The denominator includes patients with an active patient status in the immunization information system as of June 1, 2024. Patient active status in an immunization information system establishes a classification of individual patients within a health care organization. Health care providers are responsible for vaccinating patients with an active status within their clinic population or geographic catchment area. Patient status is changed to inactive when the patient changes providers, moves, or is lost to follow-up, or deceased if patient death is confirmed through manual review or system linkage with vital statistics or other health records. https://repository.immregistries.org/files/resources/5835adc2dad8d/mirow_pais_mini-guide.pdf

^†^ Hib primary series = receipt of ≥2 or ≥3 doses, depending on product type received; full series = primary series and booster dose, which includes receipt of ≥3 or ≥4 doses, depending on product type received.

^§^ The combined six-vaccine series (4:3:1:3*:3:4) includes ≥4 doses of DTaP, ≥3 doses of poliovirus vaccine, ≥1 dose of measles-containing vaccine, the full Hib series (≥3 or ≥4 doses, depending on product type), ≥3 doses of HepB, and ≥4 doses of PCV.

## Discussion

Vaccination coverage of ≥90% by age 24 months for vaccines included in USAPI immunization programs has been inconsistently met, and coverage was substantially lower than U.S. national estimates (*5*). USAPI immunization program staff members have cited the following barriers to vaccine delivery and vaccination coverage: lack of access to reliable transportation for vaccine delivery to remote populations, lack of technical expertise to create data-driven vaccination delivery outreach plans, and issues related to governance and release of timely funding to support activities, among other barriers (*6*,*7*). Coverage as of June 1, 2024, indicates that catch-up vaccination occurred after age 24 months, particularly as children reached school entry age. Annual analyses of age at vaccination suggest that delays in initiating and completing recommended vaccination series on time have been common (A Tippins, CDC, unpublished data, 2021–2024). This phenomenon might be further evidenced by this assessment, indicated by large increases in coverage assessed after age 24 months. Gaps in immunization coverage identified across USAPI can be used to pinpoint where further efforts are needed to evaluate the jurisdiction-specific reasons for, and ways to improve, on-time vaccination.

Differences in funding among jurisdictions might affect vaccine access and program operations; however, empiric research on facilitators and barriers specific to each jurisdiction is lacking. Further, the COVID-19 pandemic might have had an additional negative impact on vaccination coverage. Coverage with most vaccines among children reaching age 24 months during 2020 (i.e., born in 2018) was higher than that among those reaching age 24 months after 2020. Existing challenges with adherence to the recommended schedule might have been amplified because of the pandemic, particularly as limited human resources were further stretched to mitigate the pandemic threat.

Catch-up campaigns are recommended to address existing lags in coverage among children born during 2017–2021.^§§§^ However, although catch-up campaigns increase coverage in the short term, mathematical models indicate that reaching and maintaining optimal levels of vaccination coverage through routine access to vaccination services is a more cost-effective long-term strategy (*8*). Evidence-based approaches to increasing vaccination coverage include strong health care provider recommendations, advocating for vaccines at every opportunity, and use of reminder and recall notices (*9*). In jurisdictions with remote populations, additional strategies related to increasing the frequency of vaccine delivery services to outer islands need to be considered. Further, lessons learned from recent successful vaccination campaigns, such as the COVID-19 vaccine rollout, can be applied to routine vaccination services to improve vaccine access and coverage (*6*,*7*).

### Limitations

The findings in this report are subject to at least three limitations. First, accuracy of coverage estimates depends on completeness and accuracy of jurisdictional IIS data. Evaluations conducted since 2016 have found high levels of completeness and accuracy of vaccination data across the five USAPI IISs (i.e., dose dates and product types between paper and IIS records matched [A Tippins, CDC, unpublished data, 2016–2023]). Second, the active patient population size in IISs can be inflated compared with census estimates because of difficulties tracking out-migration and deaths. These difficulties can lead to an underestimation of vaccination coverage. Recent census data were not available for denominator estimation for all jurisdictions included in this assessment. For this reason, the Modeling of Immunization Registry Operations Workgroup exclusion criteria (*4*) were applied to classify likely active-patient status of patients in IIS. Finally, vaccination coverage for Guam is assessed via the National Immunization Survey and was not included in this analysis (*10*). Differences in vaccination coverage estimation methods might mean that results for Guam are not directly comparable with IIS-based estimates for the other USAPI presented in this report.

### Implications for Public Health Practice

Vaccination coverage data can support evaluation of activities by USAPI immunization programs and their partners to increase on-time vaccination of children by age 24 months. The gaps in immunization coverage identified in this report can be used in future research and evaluation to systematically identify determinants of on-time vaccination by jurisdiction. Qualitative research, such as key informant interviews with immunization program stakeholders, might help identify facilitators and barriers to immunization program operations by jurisdiction and to examine the feasibility of implementing evidence-based interventions to improve vaccination service delivery while mitigating identified barriers. Continued monitoring of vaccination coverage can be used to guide intervention implementation and evaluate effectiveness of interventions.

## Footnotes

* The U.S. Healthy People 2030 and WHO’s Immunization Agenda 2030 vaccination objectives include ≥90% coverage by age 2 years with ≥1 dose of the measles, mumps, and rubella vaccine and ≥4 doses of diphtheria and tetanus toxoids and acellular pertussis vaccine. WHO’s Immunization Agenda 2030 goals also include an objective of ≥90% coverage with ≥3 doses of pneumococcal conjugate vaccine.

† USAPI comprises three U.S. territories (American Samoa, Guam, and Northern Mariana Islands) and three freely associated states (Federated States of Micronesia, Marshall Islands, and Palau). All jurisdictions receive Section 317 Immunization Program funding, which is a discretionary program to purchase vaccines and support immunization infrastructure. The U.S. territories also receive funding through the Vaccines for Children program, which is an entitlement program that provides vaccines at no cost to eligible children and adolescents aged ≤18 years through enrolled health care providers.

§ Jurisdictions reviewed in this report include American Samoa, Northern Mariana Islands, Federated States of Micronesia, Marshall Islands, and Palau. Vaccination coverage among children in Guam has been assessed via the National Immunization Survey since 2013; IIS-based coverage assessment was not conducted for Guam. Information on childhood vaccination coverage in Guam is available online. https://www.cdc.gov/vaccines/imz-managers/coverage/childvaxview/interactive-reports/index.html

¶ Patient active status in an IIS establishes a classification of individual patients within a health care organization. Health care providers are responsible for vaccinating patients with an active status within their clinic population or geographic catchment area. Patient status is changed to inactive when the patient changes providers, moves, or is lost to follow-up, or deceased if patient death is confirmed through manual review or system linkage with vital statistics or other health records. https://repository.immregistries.org/files/resources/5835adc2dad8d/mirow_pais_mini-guide.pdf

** The combined six-vaccine series (4:3:1:3*:3:4) includes ≥4 doses of DTaP; ≥3 doses of poliovirus vaccine; ≥1 dose of measles-containing vaccine; ≥3 or ≥4 doses (depending upon product type) of Hib; ≥3 doses of HepB; and ≥4 doses of PCV.

†† American Samoa and Northern Mariana Islands have included HepA and varicella vaccine in their vaccination programs. The Federated States of Micronesia, Marshall Islands, and Palau recommend the second dose of MMR 4 weeks after receipt of the first dose, which is recommended at age 12 months.

§§ 45 C.F.R. part 46, 21 C.F.R. part 56; 42 U.S.C. Sect. 241(d); 5 U.S.C. Sect. 552a; 44 U.S.C. Sect. 3501 et seq.

¶¶ Hib primary series = receipt of ≥2 or ≥3 doses, depending on product type received; full series = primary series and booster dose, which includes receipt of ≥3 or ≥4 doses, depending on product type received.

*** The birth dose of HepB is measured as the proportion of children who received a dose of HepB by age 3 days.

††† Includes ≥2 doses of Rotarix monovalent rotavirus vaccine (GSK) or ≥3 doses of RotaTeq pentavalent rotavirus vaccine (Merck and Co., Inc.); if any dose in the series is either RotaTeq or unknown, the default is to a 3-dose series. Rotavirus is assessed at age 8 months to reflect the maximum age at administration.

§§§ Recommendation consistent with WHO’s The Big Catch Up: Essential Immunization Recovery Plan for 2023 and Beyond. https://iris.who.int/bitstream/handle/10665/371801/9789240075511-eng.pdf?sequence = 1
